# Management of a Staged Approach in an Implant‐Supported Complete‐Arch Fixed Dental Prosthesis (ISCFDP) With a Long Term, Long Span Fixed Provisional Prosthesis, Leveraging a Full Digital Approach: A Case Report

**DOI:** 10.1111/jerd.70049

**Published:** 2025-10-23

**Authors:** Kiarash Karimi, Carlos A. Jurado, Masoud Hassan Zadeh, Toshiki Nagai

**Affiliations:** ^1^ Private Practice Woodland Hills California USA; ^2^ Section of Restorative Dentistry, Division of Preventive and Restorative Sciences UCLA School of Dentistry Los Angeles California USA; ^3^ Division of Operative Dentistry, Department of General Dentistry University of Tennessee Health Science Center College of Dentistry Memphis Tennessee USA; ^4^ Ponce Health Sciences University School of Dental Medicine Ponce Puerto Rico; ^5^ Private Practice Drachten Friesland the Netherlands; ^6^ Department of Prosthodontics Indiana University School of Dentistry Indianapolis Indiana USA

**Keywords:** digital workflow, full‐arch rehabilitation, implant‐supported prosthesis, ISCFDP, photogrammetry, staged approach

## Abstract

**Objectives:**

To describe the clinical implementation of a staged approach using strategically selected abutment teeth, a cobalt‐chromium‐reinforced long‐span provisional prosthesis, and a fully digital workflow for maxillary FP‐2 full‐arch rehabilitation.

**Overview:**

A 48‐year‐old male with advanced periodontal disease underwent a staged full‐arch implant rehabilitation. Maxillary canines and first molars were preserved to support a long‐span fixed provisional prosthesis. A fully digital workflow was employed, including CBCT, intraoral scanning, and photogrammetry. A cobalt‐chromium framework reinforced the provisional prosthesis, enhancing strength and stability during the healing period. Following soft tissue maturation, a definitive zirconia prosthesis was digitally designed and delivered with minimal adjustment.

**Conclusion:**

This case highlights the value of a strategically staged approach that preserves key abutment teeth and incorporates a robust digital workflow. The method enabled precise implant placement, improved prosthetic predictability, and optimized esthetic and functional outcomes.

## 1 Introduction

Although the global prevalence of edentulism is decreasing, the growing life expectancy has led to a sustained need for dental rehabilitation among edentulous patients [1]. Immediate loading of implant‐supported complete‐arch fixed dental prostheses (ISCFDPs) has emerged as a favored treatment modality, providing rapid esthetic improvement, functional restoration, and less postoperative discomfort compared to traditional methods [2, 3]. According to Misch's classification, ISCFDPs are categorized into three types: FP‐1 (replacing only the missing teeth), FP‐2 (featuring overcontoured tooth replacements), and FP‐3 (substituting both teeth and soft tissue) [4]. The selection of prosthesis is tailored to the patient's individual anatomical characteristics, taking into account factors such as the position of the maxillary central incisors, lip mobility, facial support, and the configuration of the edentulous ridge—collectively assessed through the lip‐tooth‐ridge (LTR) classification [5]. Alveolar ridge reduction is generally recommended when additional prosthetic space is needed or to conceal the transition line between the prosthesis and the soft tissue [6]. However, in maxillary edentulous cases, post‐extraction healing frequently produces a flat ridge, making it difficult to achieve a predictable scalloped soft tissue profile. This limitation is especially significant in FP‐1 and FP‐2 prostheses and in LTR Class I‐HER (high esthetic risk) cases, where increased lip mobility tends to reveal the prosthetic–tissue junction [5, 6, 7]. To minimize the issues, advancements in digital technology have revolutionized prosthetic‐driven implant planning, especially for edentulous patients.

With no significant difference in implant survival rates across loading protocols for ISCFDPs [8, 9], immediate interim ISCFDPs have become a popular treatment option [10, 11, 12, 13]. However, a staged approach may be preferred in cases requiring significant alveolar reduction to accommodate an FP‐3 design for immediate loading, particularly when esthetic concerns, sinus proximity, hygiene access, or a history of periodontal disease present long‐term risks [14, 15, 16].

This patient report presents a staged full‐arch rehabilitation using a digitally fabricated cobalt‐chromium (Co‐Cr)‐reinforced provisional prosthesis, retained on strategically preserved teeth. A customized tooth‐supported surgical guide enabled precise implant placement within a fully digital workflow. The use of photogrammetry and intraoral scanning protocols allowed for the sequential fabrication of a milled polymethyl methacrylate (PMMA) prototype and a definitive zirconia prosthesis, ensuring optimal function, esthetics, and hygiene over the treatment course.

## 2 Clinical Case Report

### 2.1 Initial Exam and Clinical Findings

**FIGURE 1:**
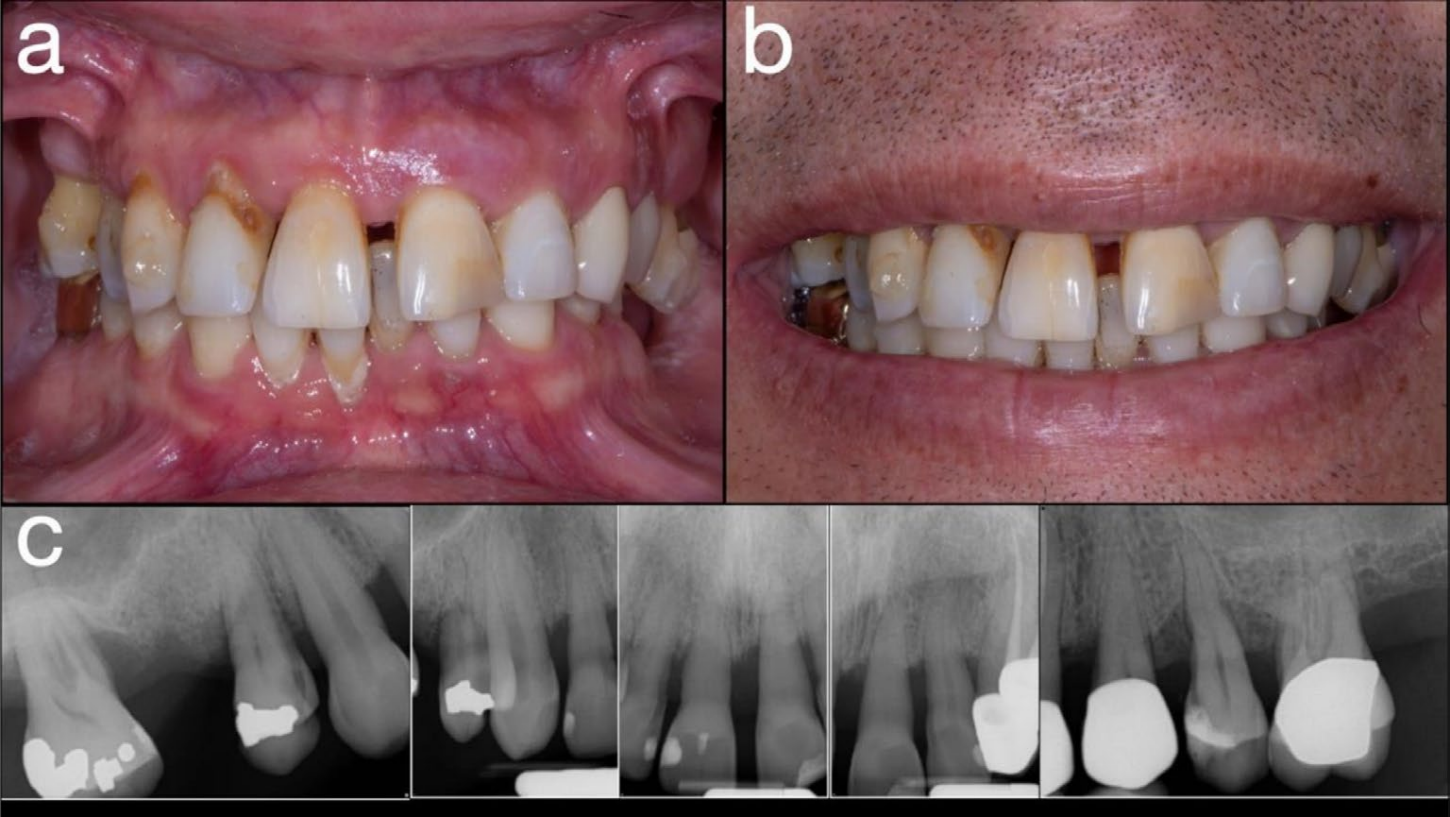
(a) Frontal view in situ; (b) smile view in situ; and (c) periapical radiograph of the maxillary dentition.

A 48‐year‐old male with a history of advanced periodontal disease presented with a desire to replace his failing maxillary dentition (written informed consent was obtained for all diagnostic, therapeutic, and publication purposes) (Figure 1). The patient was a systemically healthy, non‐smoking individual with no history of diabetes, cardiovascular disease, or bruxism. He reported no regular medications and denied any parafunctional habits. Six months before presentation, he had undergone non‐surgical periodontal therapy and demonstrated good compliance with oral hygiene instructions. At the initial examination, residual periodontal pockets ranged from 4 to 6 mm with generalized attachment loss exceeding 50%. Tooth mobility Grades II–III were observed in the maxillary dentition. Prior to surgical intervention, the patient received professional prophylaxis and reinforcement of plaque control to minimize microbial load and promote optimal healing. Clinical and radiographic evaluation revealed generalized severe chronic periodontitis, pathologic tooth mobility, vertical dimension collapse, and supraeruption of posterior teeth. The prognosis of all remaining maxillary teeth was determined to be hopeless [17]. The patient declined removable prosthetic options; therefore, a staged treatment approach was planned, involving a long‐term fixed provisional prosthesis followed by a definitive zirconia ISCFDP.

### 2.2 Treatment Planning

**FIGURE 2:**
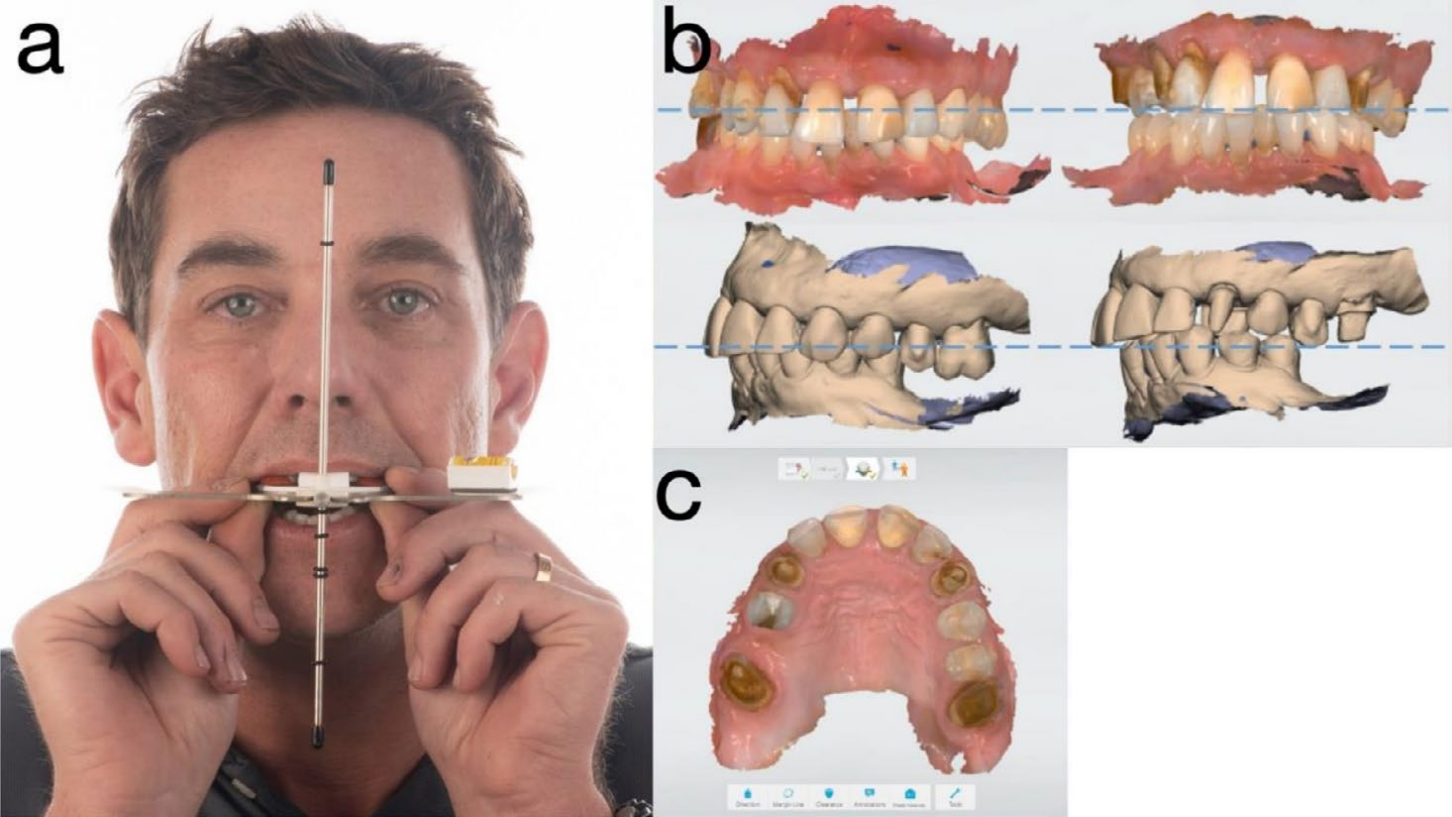
(a) Dental facial analyzer (DFA) used for maxillary orientation; (b) intraoral scan (IOS) at maximum intercuspation (MIP) and at increased occlusal vertical dimension (OVD); and (c) IOS occlusal view following preparation of maxillary canines and first molars.

Due to the high risk of complications associated with immediate loading—including the need for extensive alveolar bone resection, risk of sinus involvement, loss of vestibular depth, and future peri‐implantitis in a periodontally compromised patient—a staged approach was chosen. A fixed provisional was planned, performing selective extractions and retention of strategic abutment teeth (maxillary canines and first molars) to support a long‐span provisional prosthesis. A fully digital workflow was employed, including cone beam computed tomography (CBCT), intraoral scan (IOS) with a calibrated intraoral scanner (TRIOS 4; 3Shape A/S, Copenhagen, Denmark), and virtual patient planning (Figure 2a).

Bilateral maxillary canines and first molars were prepared, and a digital impression was obtained using a calibrated intraoral scanner (TRIOS 4; 3Shape A/S, Copenhagen, Denmark). Due to a loss of vertical dimension of occlusion, supra‐eruption of the maxillary left posterior teeth, and in consideration of facial esthetics (dropping mouth corners), interocclusal rest space (8‐10 mm), speaking space (6‐8 mm at anterior region), excessive vertical overlap and the proposed incisal edge position, it was determined that the occlusal vertical dimension (OVD) should be increased by 6 mm in the anterior region. This new OVD was established using a Lucia jig, and a occlusal registration was subsequently recorded at this position [18] (Figure 2b,c). The patient was monitored for temporomandibular joint comfort and masticatory muscle adaptation during a 4‐week provisionalization period, during which no muscle tenderness, joint sounds, or discomfort were reported. The patient exhibited satisfactory adaptation to the new mandibular position before proceeding with the next stage.

**FIGURE 3:**
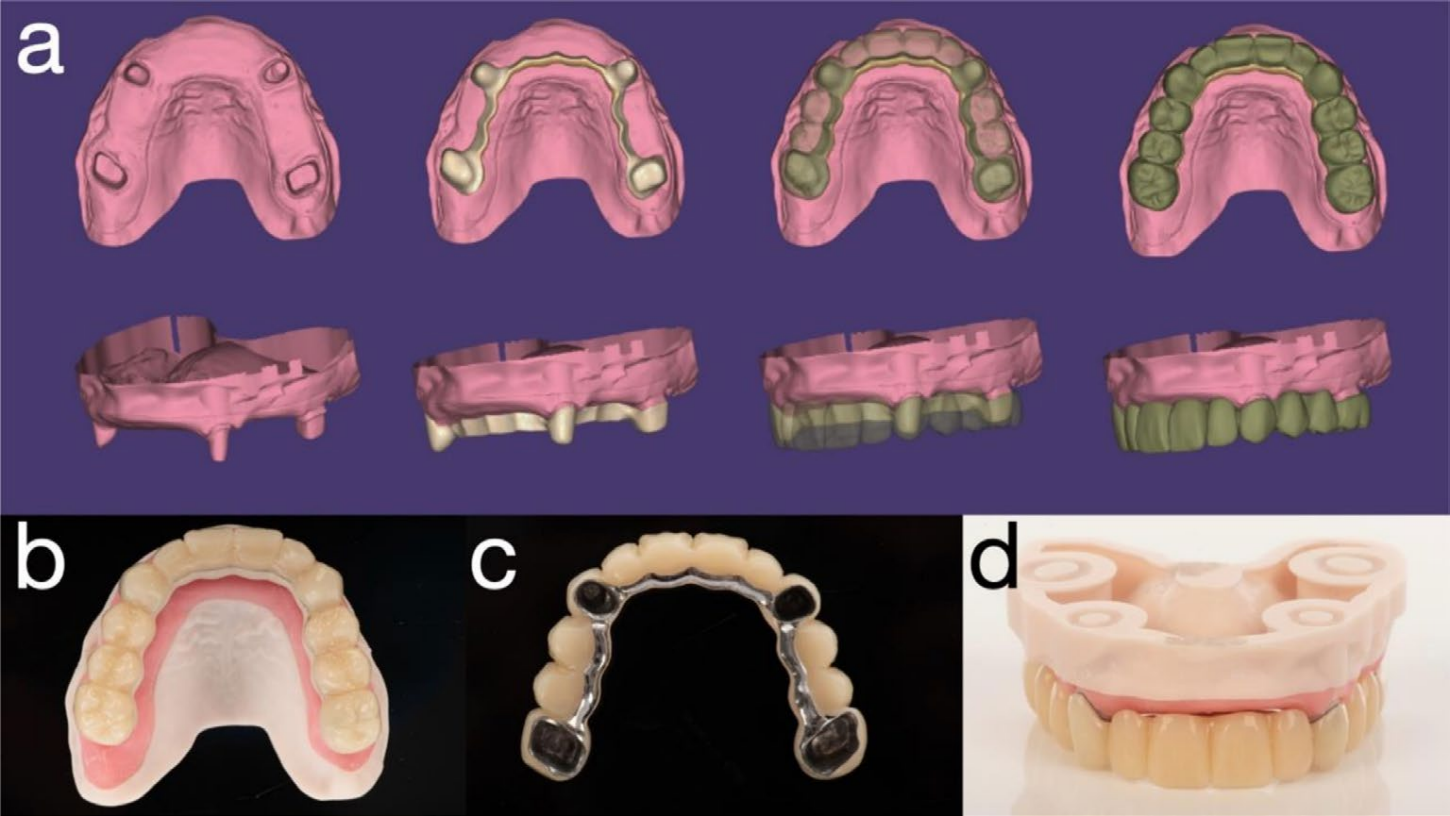
(a) Fixed provisional prosthesis designed using computer‐aided design (CAD) software (exocad; exocad GmbH, Darmstadt, Germany); (b) occlusal surface; (c) intaglio surface; and (d) frontal view of the provisional prosthesis.

**FIGURE 4:**
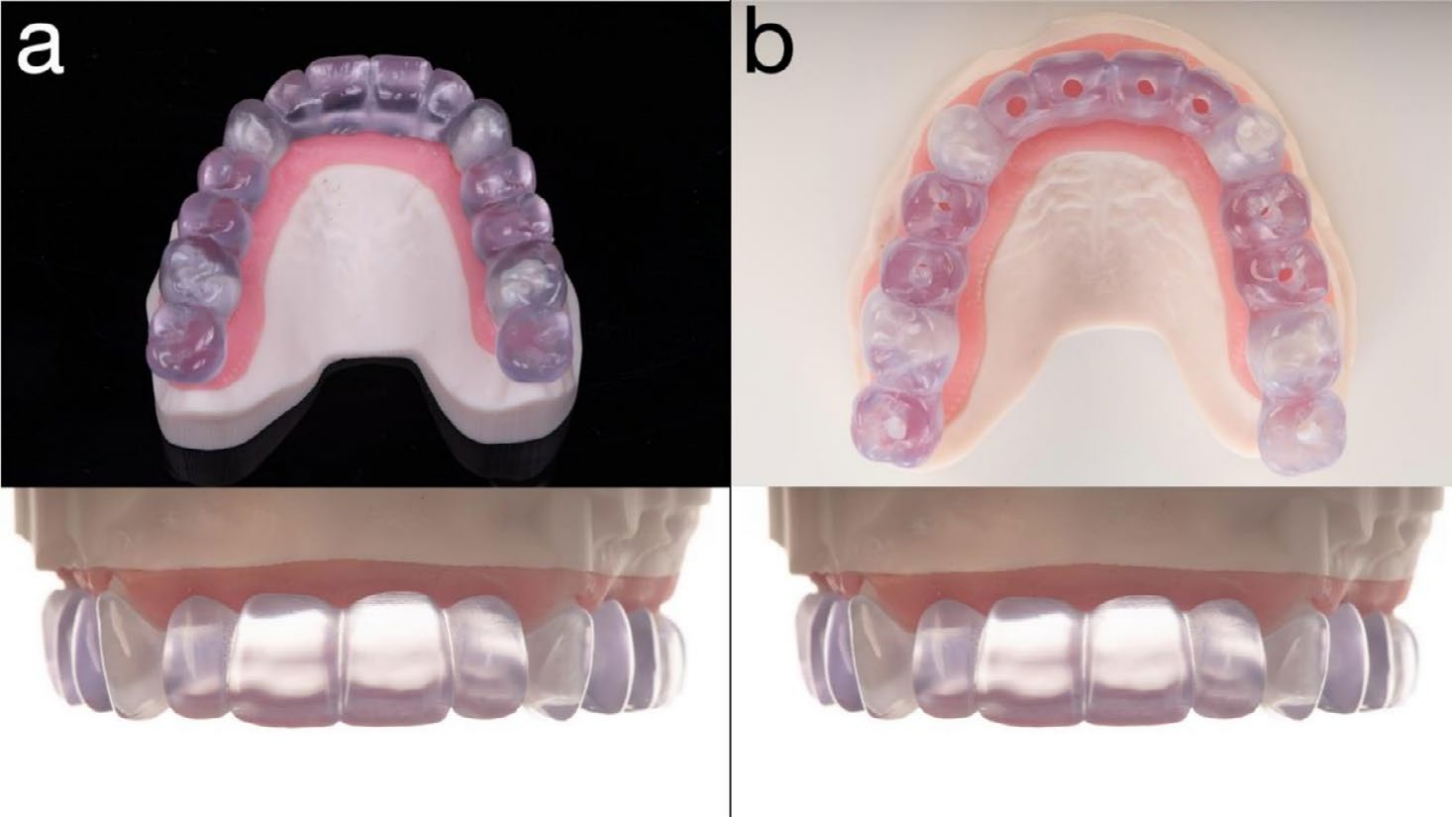
(a) Radiographic stent and (b) surgical stent used for guided implant placement.

Using computer‐aided design (CAD) software (exocad; exocad GmbH, Darmstadt, Germany), the remaining anterior teeth (incisors and premolars) were digitally removed, and a fixed provisional prosthesis—retained by the maxillary first molars and canines—was designed based on a digital diagnostic wax‐up (Figure 3a). Given the extended span of the prosthesis and limited restorative space, a novel approach was employed to enhance structural integrity by incorporating a Co‐Cr substructure (Figure 3b–d). To facilitate fabrication, the digital wax‐up was separated into two distinct files: one for the Co‐Cr framework and the other for the overlying PMMA superstructure. Co‐Cr was selected due to its superior flexural strength and high machinability, making it suitable for long‐span provisional restorations [19]. The Co‐Cr framework was milled using a five‐axis milling machine (Roders GmbH, Germany), and the PMMA prosthesis was fabricated using a five‐axis milling unit (PrograMill PM7; Ivoclar Vivadent, Schaan, Liechtenstein). For improved esthetics, the lingual surface of the metal framework was coated with a porcelain opaquer (Noritake, Tokyo, Japan). As bonding between PMMA and metal is inherently unpredictable, surface pretreatment was conducted to optimize adhesion. Both the Co‐Cr framework and the intaglio surface of the PMMA structure were air‐abraded with 110 μm silica‐coated alumina particles (Rocatec Plus; 3M ESPE, Seefeld, Germany) prior to assembly. The two components were luted using a self‐adhesive universal resin cement (RelyX Unicem; 3M ESPE, Seefeld, Germany). A surgical guide and a radiographic stent were fabricated using the same STL dataset derived from the digital planning workflow and fabricated using a biocompatible resin (KeyGuide; Keystone Industries, Gibbstown, NJ, USA) and printed with a UV light‐curing 3D printer (Asiga Max UV; Asiga, Alexandria, Australia) (Figure 4a,b).

### 2.3 Surgical Phase and Provisionalization

**FIGURE 5:**
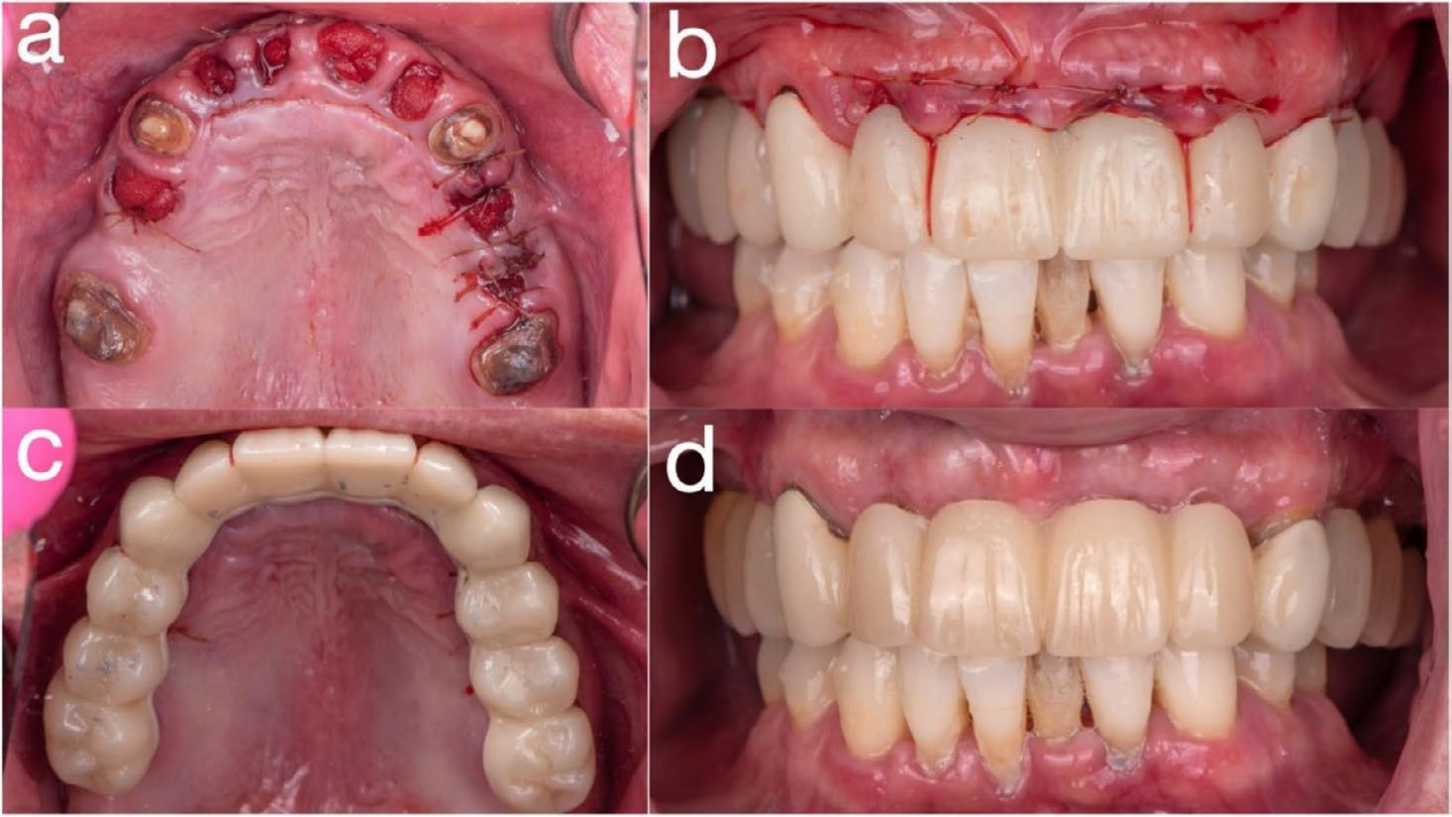
(a) Extraction of all non‐abutment maxillary teeth; (b) frontal view immediately post‐extraction with fixed provisional prosthesis in place; (c) occlusal view post‐extraction with fixed provisional prosthesis; and (d) frontal view at 2‐week follow‐up with fixed provisional prosthesis.

**FIGURE 6:**
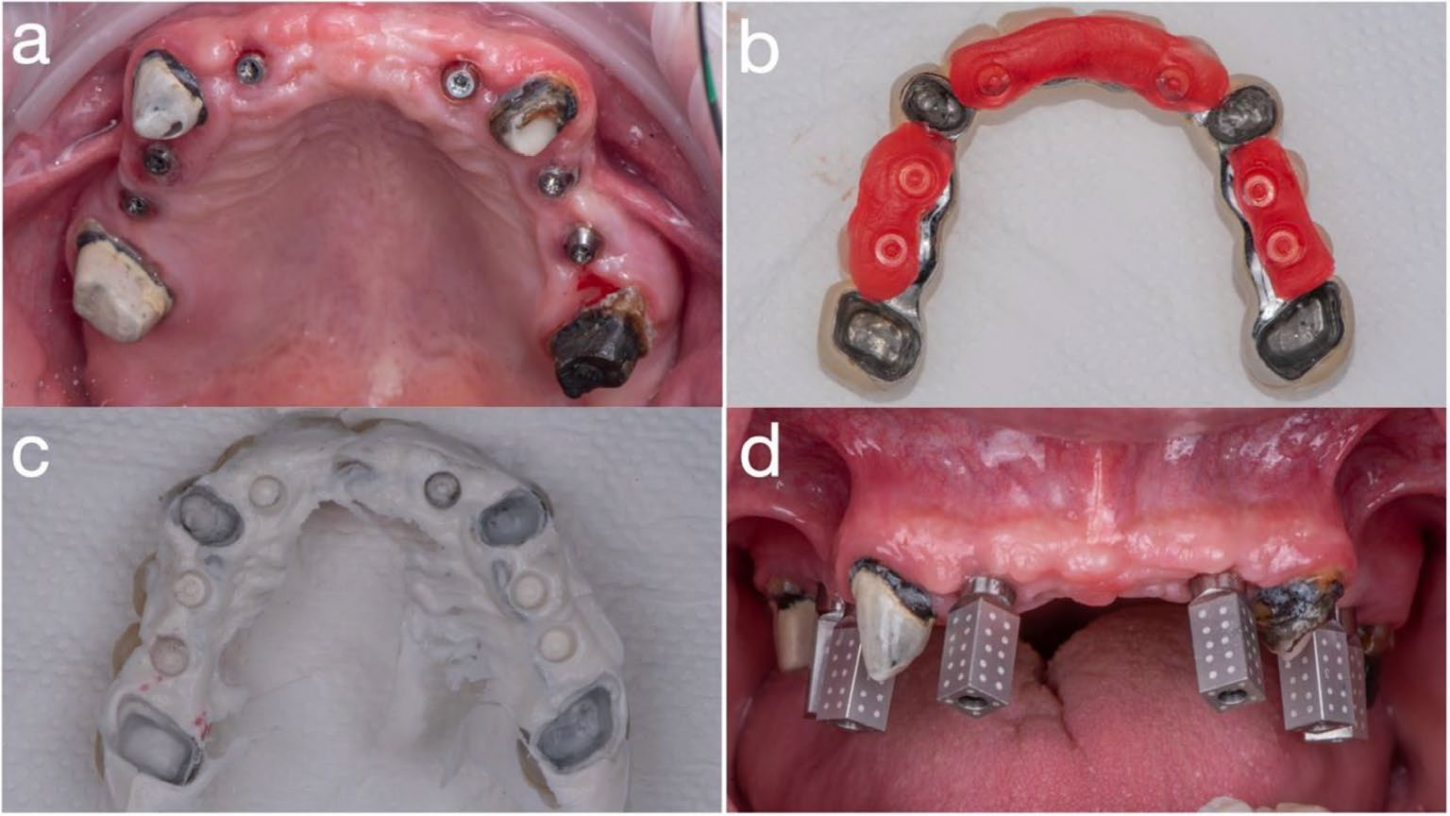
(a) Placement of six dental implants; (b) red periphery wax used to evaluate and adjust the intaglio surface of the fixed provisional prosthesis; (c) fit‐checker applied to confirm intaglio adaptation; and (d) photogrammetry performed to capture three‐dimensional implant positions.

All non‐abutment maxillary teeth were atraumatically extracted, and the provisional prosthesis was immediately delivered and cemented using a high‐strength provisional resin cement (Premier Dental, Plymouth Meeting, PA, USA). Occlusion was carefully adjusted to ensure functional harmony. During the provisional phase, the prosthesis was periodically removed for hygiene maintenance, cleaned, and recemented as needed (Figure 5a–d). Four months post‐extraction, implant planning was completed using updated CBCT. Six dental implants (Straumann Bone Level Tapered [BLT], *Ø*4.1 mm × 12 mm; Institut Straumann AG, Basel, Switzerland) were placed at positions #12, #13, #14, #22, #24, and #25 using a fully guided protocol with a tooth‐supported PMMA surgical stent. Soft tissue healing was facilitated by careful design of the provisional framework to avoid impinging on the ridge. All implants achieved insertion torques between 35 and 45 N cm, indicating adequate primary stability for staged loading. Multiunit abutment (MUA) (screw‐retained abutments (SRAs); Institut Straumann AG, Basel, Switzerland) and protective caps (Institut Straumann AG, Basel, Switzerland) were placed at second‐stage surgery, and the intaglio surface of the provisional was adjusted accordingly (Figure 6a–c).

### 2.4 Digital Impression and Prototype Prosthesis

**FIGURE 7:**
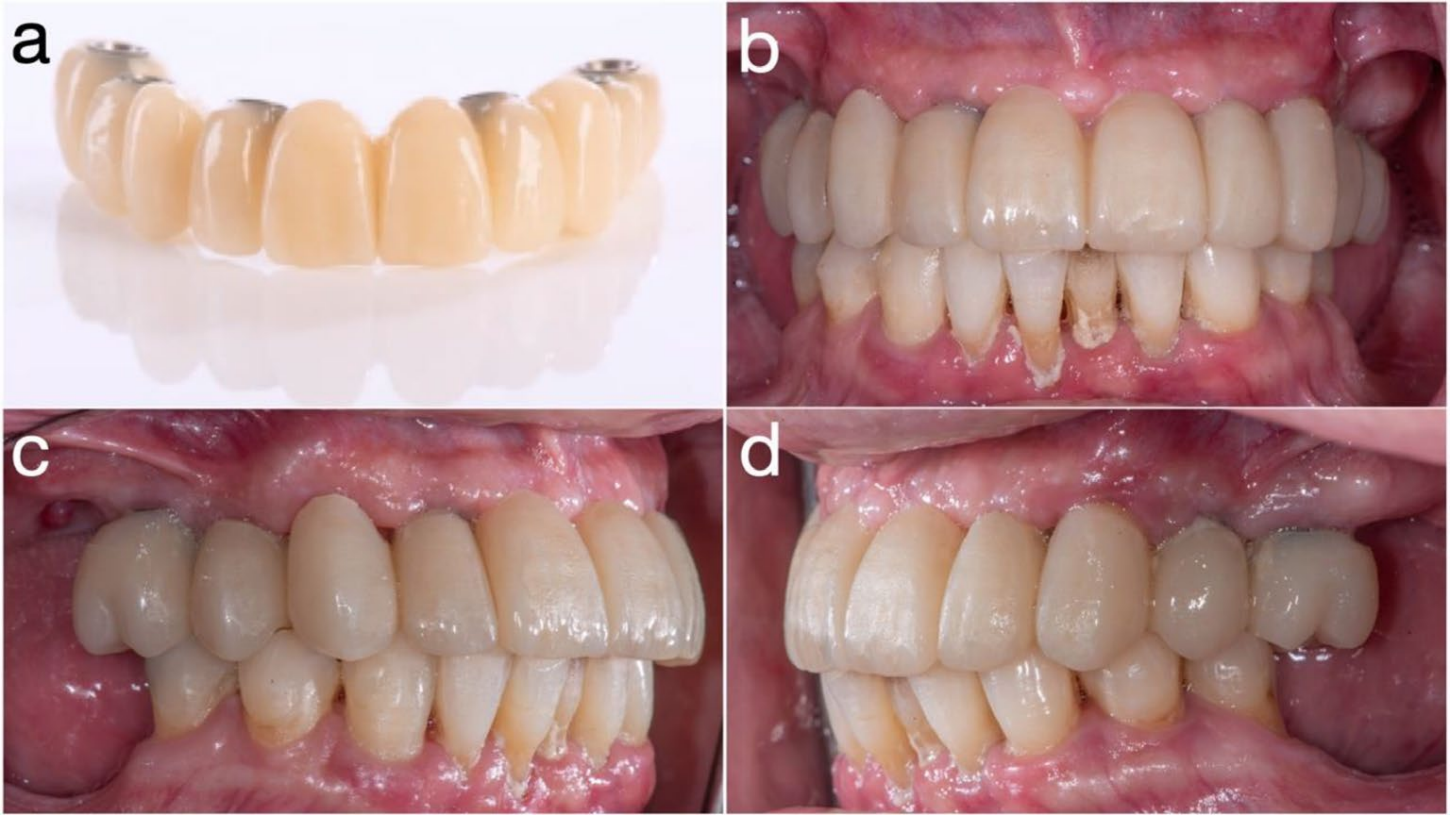
(a) Milled PMMA prototype; (b) frontal view of prototype in situ; (c) right lateral view; and (d) left lateral view of the prototype placed intraorally.

After soft tissue healing, photogrammetry (iCam4D; iMetric 3D GmbH, Courgenay, Switzerland) was used to record 3D implant positions (Figure 6d). A digital impression protocol combined scans of the existing provisional in the patient's mouth, protective caps with soft tissue, and a 360° extraoral scan of the prosthesis. These data were superimposed to design a prototype prosthesis in CAD software (exocad; exocad GmbH, Darmstadt, Germany), and fabricated in milled PMMA. The prototype was delivered following extraction of the abutment teeth (bilateral maxillary canines and first molars) and monitored for 3 months with adjustments to promote tissue maturation (Figure 7a–d).

### 2.5 Definitive Prosthesis Fabrication and Delivery

**FIGURE 8:**
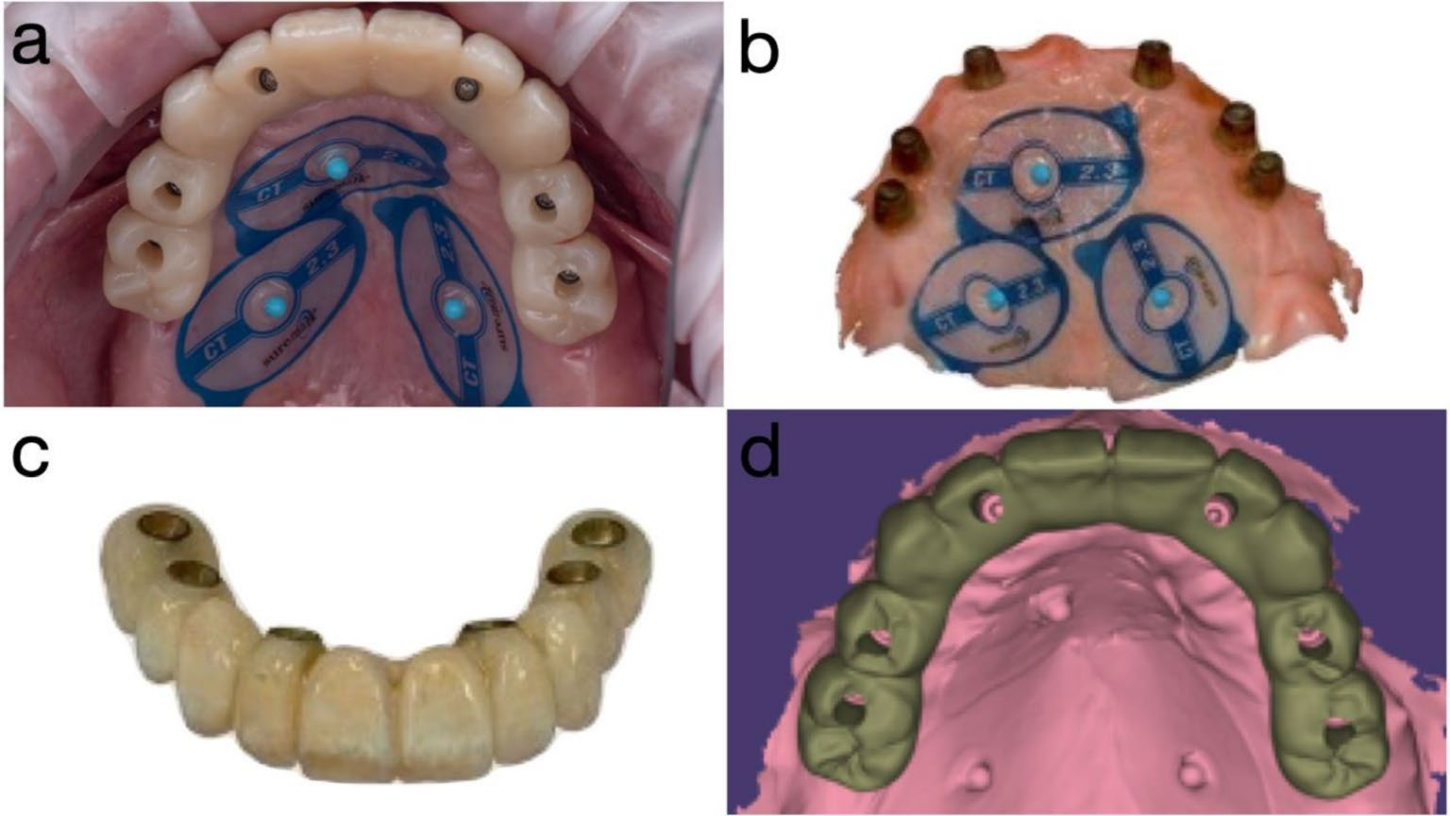
(a) Intraoral scan (IOS) of the provisional prosthesis in situ with fiducial marker; (b) IOS of soft tissue; (c) 360° IOS of the provisional prosthesis; and (d) superimposition of all datasets in computer‐aided design (CAD) software (exocad; exocad GmbH, Darmstadt, Germany).

**FIGURE 9:**
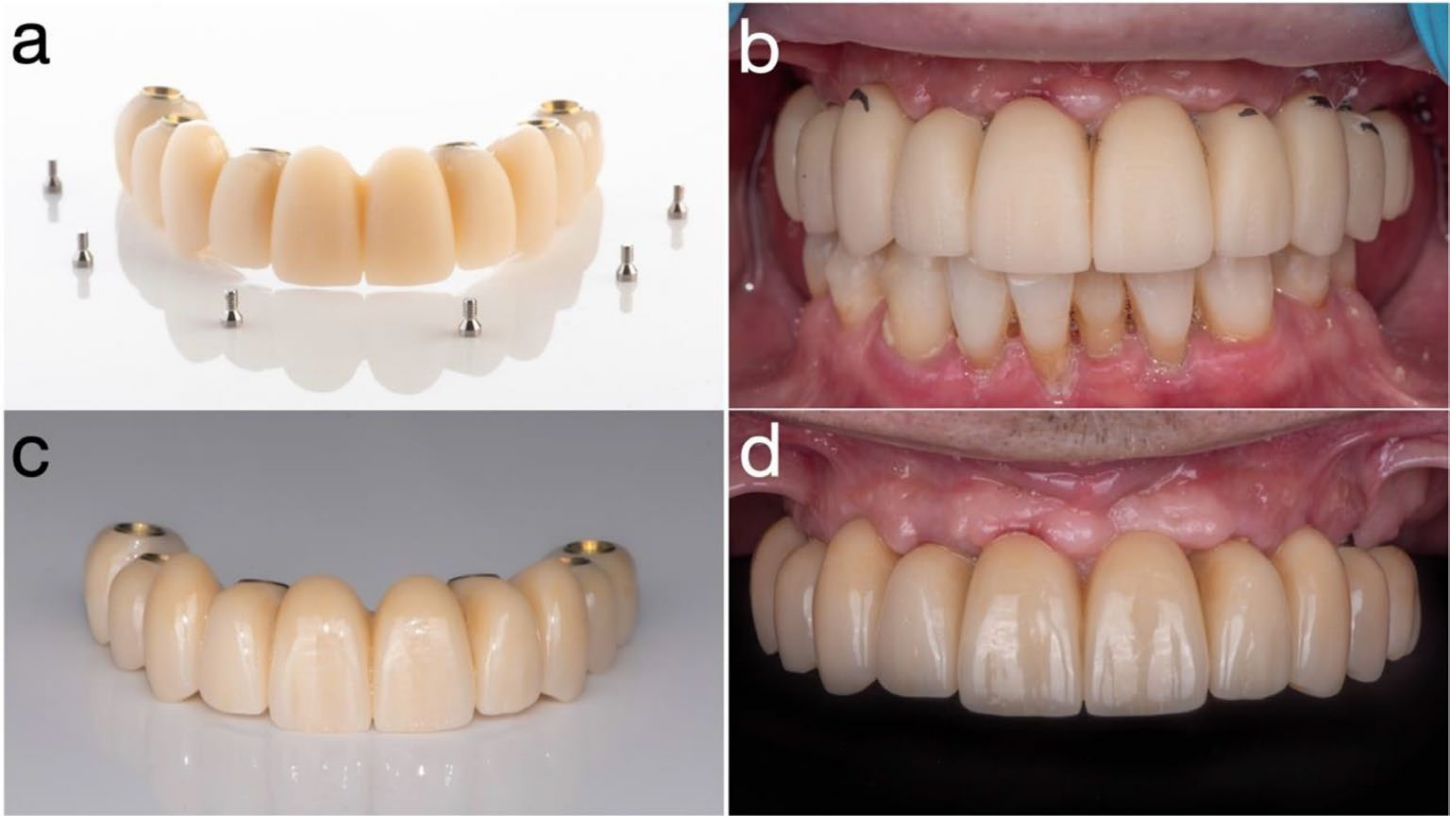
(a) Bisque‐back stage of the definitive zirconia implant‐supported complete‐arch fixed dental prosthesis (ISCFDP); (b) minor intraoral adjustments at the bisque‐back stage; (c) definitive zirconia ISCFDP; and (d) frontal view at delivery of the definitive prosthesis.

Once soft tissue contours had stabilized, new IOS datasets were acquired, including scans of the provisional prosthesis in the patient's mouth, soft tissue with protective caps, and a 360° IOS of the provisional itself. These datasets were superimposed using fiduciary markers to facilitate precise alignment in CAD software (exocad; exocad GmbH, Darmstadt, Germany) (Figure 8a–d). A definitive zirconia ISCFDP with facial cutback was designed and fabricated from multilayered zirconia (Katana Zirconia YML; Kuraray Noritake Dental Inc., Nagoya, Japan). Porcelain veneering was applied selectively in the esthetic zones. Prior to finalization, the prosthesis was tried in at the bisque‐back stage to allow for minor refinements (Figure 9a,b). The final zirconia prosthesis was extraorally bonded to titanium cylinders (Variobase for Bridge/Bar cylindrical coping) using a dual‐cure resin cement in a freehand manner, without the use of a physical cast, to create a screw‐mentable implant restoration structure. The MUAs were in turn torqued to the implants at 35 N cm. The passive fit of the definitive ISCFDP was confirmed through a combination of one‐screw test, screw‐resistance test, and periapical radiographic evaluation. The restoration was seated intraorally and tightened at a single most distal implant site to evaluate for any visible lift‐off or misfit at the remaining abutments (one‐screw test). Sequential screw tightening was then performed while monitoring for screw resistance or strain, confirming the absence of internal tension. Periapical radiographs were obtained at each implant site to verify intimate adaptation between the titanium bases and MUAs without radiographic gaps. The screw‐mentable restoration was then screw‐retained to the MUAs (SRAs; Institut Straumann AG) with a torque of 15 N cm, according to the manufacturer's recommendation. The final ISCFDP was delivered with minimal occlusal adjustment, exhibiting excellent esthetic integration and favorable cleansability (Figure 9c,d).

### 2.6 Postoperative Management and Follow‐Up

**FIGURE 10:**
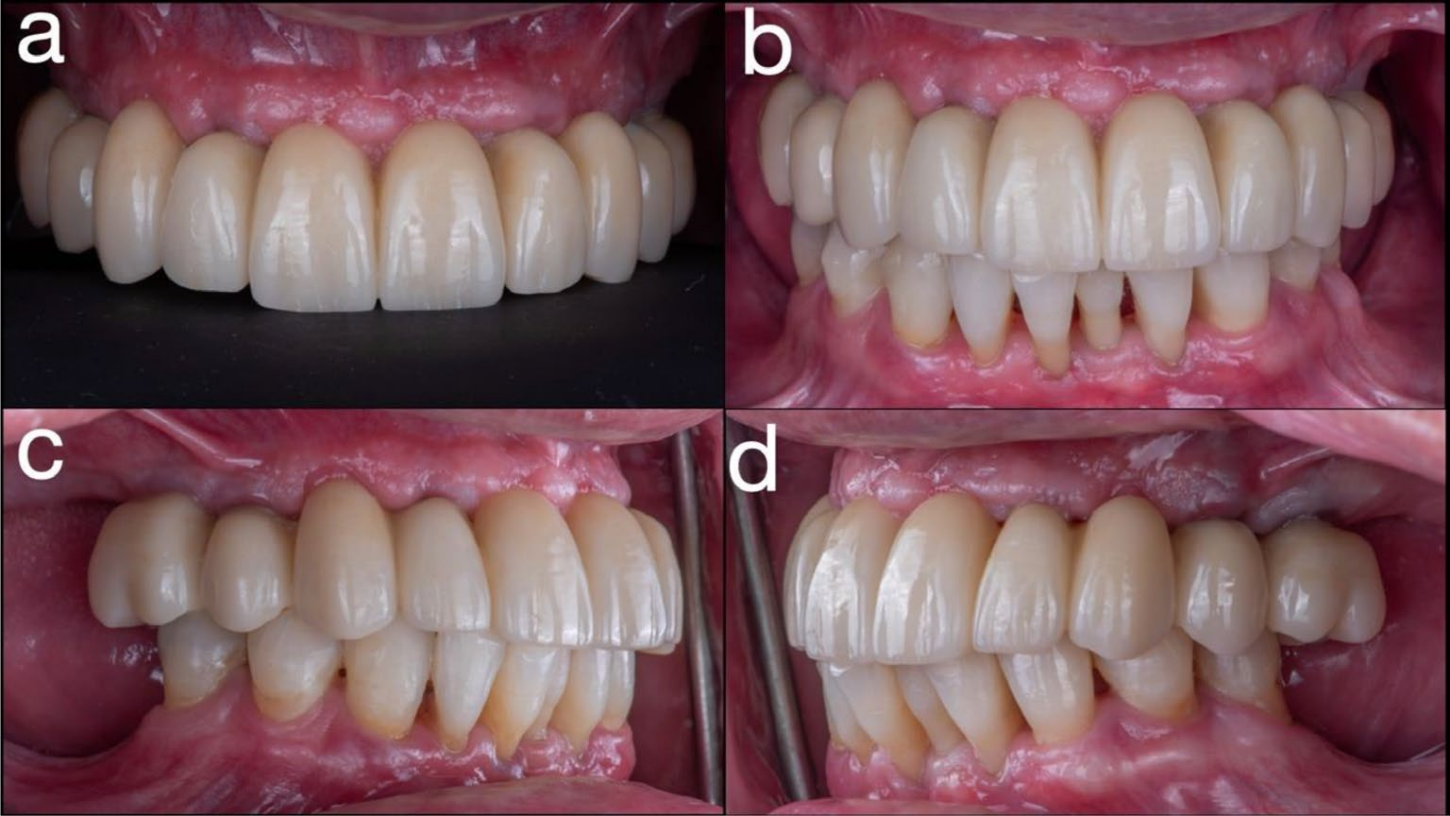
(a) Intraoral view at 2‐year follow‐up; (b) frontal view; (c) right lateral view; and (d) left lateral view at 2‐year follow‐up.

**FIGURE 11:**
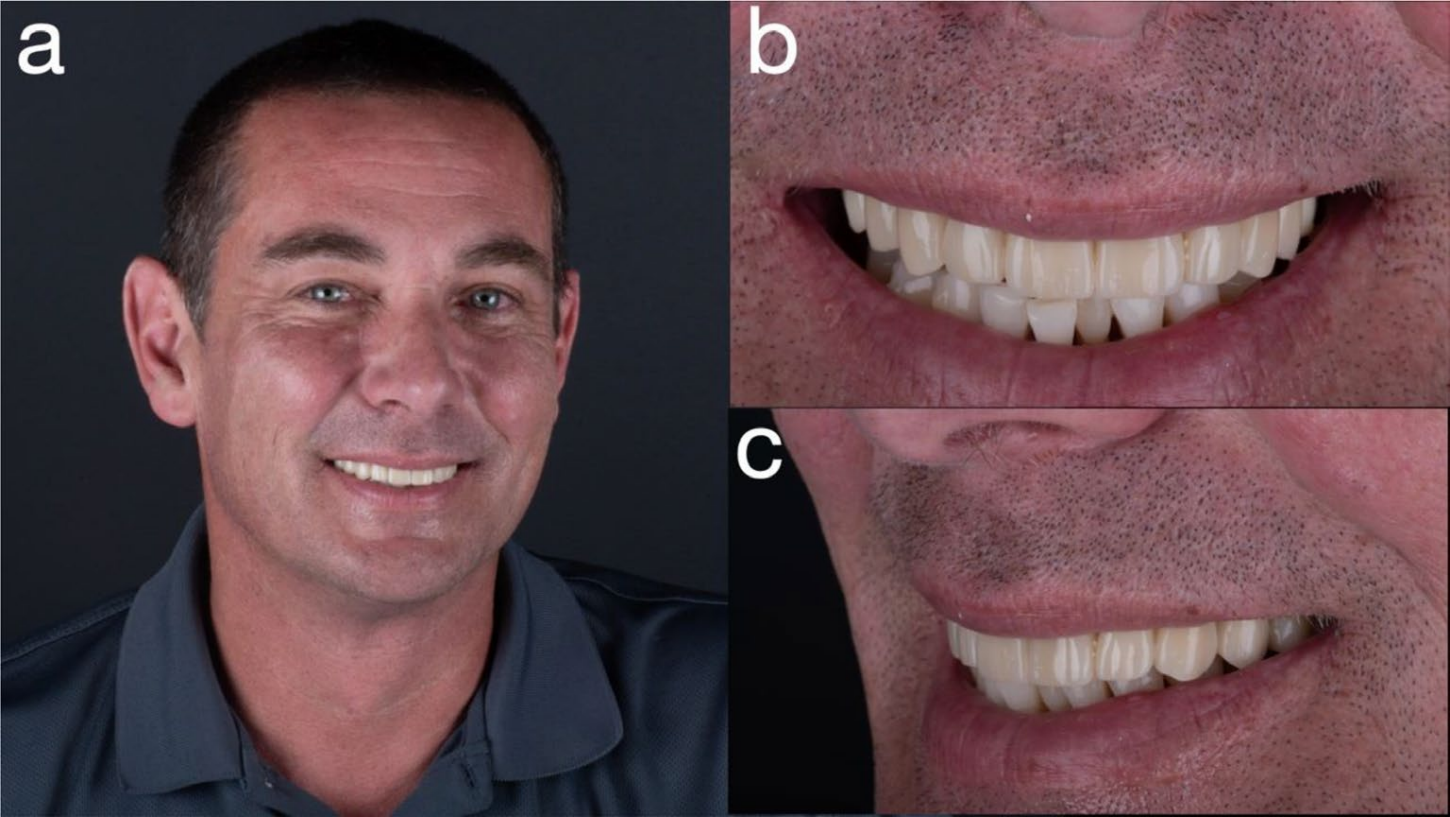
(a) Extraoral photograph at 2‐year follow‐up; (b) frontal view of smile; and (c) left lateral view of smile.

A protective occlusal splint (BioSplint; Dental Direkt GmbH, Spenge, Germany) was milled from a PMMA‐based resin using a five‐axis milling machine (Arum 5X‐450; Doowon, Daejeon, South Korea) 1 week postdelivery and adjusted for centric occlusion and anterior guidance. Hygiene maintenance was provided at 1‐, 3‐, and 6‐month intervals during the first postoperative year and every 6 months thereafter. Professional maintenance included air polishing with erythritol–chlorhexidine powder and evaluation of peri‐implant tissues and prosthetic components. At each visit, peri‐implant probing depths remained ≤ 4 mm with no bleeding on probing, and radiographs demonstrated stable crestal bone levels. No mechanical or biological complications—such as screw loosening, veneer chipping, or peri‐implant inflammation—were observed throughout the 2‐year follow‐up period (Figures 10 and 11). The intraoral photographs presented in Figures 5 and 7 were captured during the provisional phase prior to professional maintenance visits, which explains the transient plaque deposits visible on the remaining natural teeth and prosthetic surfaces. Subsequent prophylaxis and reinforced oral‐hygiene instruction were provided, resulting in the excellent peri‐implant tissue health demonstrated in Figures 10 and 11.

At the 2‐year follow‐up, the patient reported sustained satisfaction with esthetics, comfort, phonetics, and hygiene accessibility.

## 3 Discussion

This patient report illustrates how a carefully planned staged approach, combined with advanced digital tools and materials, can lead to predictable, esthetic, and functional outcomes in FP‐2 ISCFDPs. The integration of a Co‐Cr‐reinforced long‐span provisional prosthesis, photogrammetry, and intraoral scanning supported precise implant placement, prosthesis design, and tissue management throughout the treatment phases.

While immediate loading has become a widely accepted option for full‐arch implant rehabilitation [8, 9, 10, 11, 12, 13], certain clinical scenarios—particularly those involving periodontal compromise, vertical dimension loss, and the need for extensive alveoloplasty—may benefit from a staged protocol. Previous reports on staged approaches have described retaining strategic teeth or mini‐implants placement as provisional abutments, followed by initial implant placement and serial extraction of the remaining teeth [16, 20]. Alternatively, some protocols emphasize selective retention of teeth during the planning phase, allowing for full‐arch implant placement in a single surgical procedure to minimize overall invasiveness [21]. However, in many cases, the number or condition of the remaining teeth may be insufficient to support a fixed provisional prosthesis alone [22]. In such situations, additional support from resilient anatomical structures—such as the tuberosity and retromolar pad—may be required to enhance prosthesis stability during the healing phase [23]. In this patient, retaining strategic abutment teeth for provisionalization allowed for optimal soft tissue healing and occlusal stabilization before implant placement. This not only reduced surgical complexity but also maintained esthetics and patient comfort during the transition period.

When selecting abutment teeth for a staged approach, considerations include their location, condition, and influence on prosthetic design [14]. Ideally, abutment teeth should be well distributed to support a fixed provisional prosthesis without cantilevers and must not interfere with future implant sites. Structurally sound teeth are preferred; any periapical pathology should be addressed promptly to prevent complications with grafting or implant placement. While periodontally involved teeth pose risks, splinting them within a fixed provisional has shown acceptable short‐term performance [24]. Restorative space and span length are critical, as they determine connector dimensions and biomechanical behavior. Increased span length exponentially raises deflection forces, requiring appropriately sized connectors. Cantilevers should be avoided due to their potential to cause debonding and fracture, especially in provisionally cemented restorations. In this patient report, bilateral maxillary first molars and canines were retained to support the fixed provisional, enabling implant placement in ideal positions without anatomical interference. (Figures 4 and 5).

The use of a Co‐Cr substructure in the long‐span provisional addressed the challenge of mechanical durability often encountered with extended provisional restoration in a staged approach [19]. Surface pretreatment protocols and luting techniques ensured stable adhesion between metal and PMMA components, enabling a resilient and esthetic interim solution. Furthermore, the innovative digital workflow facilitated seamless integration of photogrammetry, 360° prosthesis scans, and IOS (provisional scan and soft tissue scan), supporting the accurate design and delivery of both the prototype and definitive zirconia restorations. By leveraging this full digital approach, clinicians were able to maintain prosthetic intent throughout all phases of treatment, from diagnostic planning to surgical execution and final prosthetic delivery. This case also demonstrates that flangeless provisionalization, tissue‐stabilized prototype prostheses, and a sequence of intraoral and extraoral scans can enhance the predictability of full‐arch rehabilitation, especially when minimal bone reduction is desirable. The digital alignment of IOS datasets using fiduciary markers ensured a precise spatial relationship between implants, soft tissue, and teeth positions, reducing chairside adjustments and improving long‐term maintainability.

Nevertheless, the limitations of this approach must be acknowledged. First, this report describes a single clinical case and therefore lacks the statistical power to generalize findings. Although the digital protocols employed—particularly the photogrammetry and extraoral scan workflows—are supported by promising in vitro studies, clinical evidence regarding their reproducibility and long‐term accuracy in diverse patient populations remains limited [25, 26, 27]. Photogrammetry offers high accuracy in capturing implant positions; however, its widespread adoption is limited by high equipment costs and restricted application, as it is designed solely for recording implant locations without soft tissue or occlusal data [27]. Another limitation lies in the reliance on manual bonding steps, such as the freehand luting of titanium bases. Although the workflow was fully digital, operator variability may affect the consistency of the passive fit in the definitive restoration, reinforcing the potential value of verification casts in clinical practice [28].

Future investigations should include larger clinical cohorts to evaluate the mechanical durability, esthetic longevity, and biological response associated with digitally fabricated long‐span provisionals and definitive prostheses. Comparative studies that assess outcomes using different intraoral scanning strategies, prosthesis types, and verification workflows will help clarify best practices and improve clinical predictability.

## 4 Conclusion

The presented patient report highlights the clinical benefits of a staged approach utilizing strategically selected abutment teeth, a Co‐Cr‐reinforced long‐span provisional prosthesis, and a fully digital workflow for maxillary FP‐2 full‐arch rehabilitation. This strategy enabled precise implant placement, optimized esthetic and functional outcomes, and supported soft tissue preservation and patient comfort throughout the treatment.

## Consent

Written informed consent was obtained from the patient for all clinical procedures and for the publication of the case details and accompanying clinical and radiographic images.

## Conflicts of Interest

The authors declare no conflicts of interest.

## Data Availability

The data that support the findings of this study are available from the corresponding author upon reasonable request.
